# FPGA-Based Two-Dimensional Matched Filter Design for Vein Imaging Systems

**DOI:** 10.1109/JTEHM.2021.3119886

**Published:** 2021-10-14

**Authors:** Wenxin Xiang, Deliang Li, Jiabing Sun, Jiawei Liu, Guowei Zhou, Yuan Gao, Xiaoyu Cui

**Affiliations:** 1 College of Medicine and Biological Information EngineeringNortheastern University 27817 Shenyang 110819 China; 2 Nursing SchoolChina Medical University, Shenbei Shenyang 110122 China

**Keywords:** Field programmable gate array (FPGA), matched filter, vein extraction, vein imaging, infrared image

## Abstract

Venipuncture is a common medical procedure. The use of augmented reality-based assistive devices can improve the first puncture success rate in patients with poor vascular filling. In order to improve the image rendering quality and speed of auxiliary equipment, this study develop a two-dimensional matched filtering algorithm on a Field Programmable Gate Array (FPGA) in a near-infrared vein imaging system, which use parallel processing to offer real-time response and is designed as a small handheld portable device. A customized dorsal hand vein image library with 200 images captured from 120 participants is used to analyze the effects of convolution kernel parameters and exposure time on vascular imaging with different depths, and the correlation model between these parameters and vascular depth are constructed. We use the Tenengrad, variance, Laplace smoothness and standard deviation as evaluation indicators, and compare our algorithm with three other related studies. Experimental results show that the rendering quality of our proposed algorithm is significantly higher than other algorithms. In addition, the rendering speed of our algorithm can reach 66 fps, which is twice faster than the current fastest algorithm.

## I. Introduction

Venipuncture and intravenous injection are common medical methods in clinical treatments. In many cases, however, patients’ blood vessels are not easy to view on account of poor condition of the thickness or length of hand veins. Obesity, dark skin color and luxuriant hair are the primary influencing factors for this [1]. Besides, when wearing protective suit, goggles or surgical gloves, medical workers have difficulty giving injections quickly and accurately. Therefore, the study of venipuncture auxiliary equipment, namely vein imaging systems, which take near-infrared images of opisthenars and project the processed hand vein images back to aid in improving the visualization of vein, is promising and valuable in various medical treatments [2].

The optical characteristic of human veins is that hemoglobin in the dorsal hand veins has a better ability to absorb near infrared light than that in the surrounding tissue [3]. That is to say, color of vein in near-infrared image is darker than that of surrounding tissue. Based on this knowledge, many efforts have been made by researchers to develop practical auxiliary equipment for venipuncture. The existing vein extraction algorithms can be mainly divided into three categories [4]; one is based on image filtering and pixel segmentation, which extracts vein region directly at the image-level; and the second is line tracking method, which starts from a seed point and gradually extends along the vascular structure [5]. For instance, Jingliang Zhao et al proposed a superpixel-based chain tracking method to segment retinal vessel [6], and Amir Hajian et al used a modified repeated line tracking method for the extraction of vein patterns [7]. Thirdly, machine learning, such as classifier, has gradually been widely used in vascular segmentation and extraction. Compared with algorithms based on region iterative tracking and classifier, the matched filtering method using window convolution has low complexity when the segmentation performance is close, and has obvious parallelizable characteristics. It is easy to be implemented on hardware architecture, so it has significantly high real-time performance. In addition to these three main methods, some other theoretical methods perform well. For instance, diffuse reflectance images at three isosbestic wavelengths can be used to measure the depth and thickness of subcutaneous veins [8].Vein imaging system which applying contrast-limited adaptive histogram equalization algorithm and combining visible light reflection and near-infrared transmission is also an innovative attempt [9]. Besides, Zhang et al applied an active-contour based method with a denoising algorithm to efficiently segment finger veins from images [10].

Recognizing and separating blood vessels from opisthenar images is the core step of the overall hand vein imaging system. The former algorithms of this process are mostly performed on CPUs [11], [12]. However, considering the characteristics of medical image processing, such as large size of images and high requirements for portability in many cases such as emergencies, software-based algorithms are time-consuming and not suitable to carry around. Additionally, with the accelerated development of electronic techniques [13], image processing systems implemented on hardware architecture have become increasingly popular. In particular, the advent of FPGA-based image programming technologies leads to compact, fast and low-power hardware solutions.An algorithm based on an active contour evolution technique called pixel-level snakes, has been developed to be computed both in a hardware platform with a pixel-processor array [14] and on an FPGA with SIMD architecture [15]. While methods based on contour tracking need to artificially give the starting point, and it is easy to terminate at the blood vessel branch, that is, the effect of treating the branch point maybe poor.

Chaudhuri et al [17] proposed a Gaussian contour model according to the gray characteristics of blood vessels, and designed a two-dimensional matched filtering algorithm based on this. Hoover et al [11] added a threshold probing technique after the previous matched filtering algorithm. This technique introduced global feature to iteratively reduce the threshold to segment blood vessels with high accuracy, however this algorithm was implemented in software and the execution was slow. Koukounis et al [19] implemented Hoover’s method on the hardware architecture with reducing the amount of filter, the speed of the algorithm was increased by 90 times. However the accuracy was about 4% lower than the reported highest accuracy on hardware architecture. Savarimuthu et al [18] also implemented a hardware implementation of matched filtering, and optimized the convolution module by merging similar terms when calculating multiplication. In this paper, our algorithm directly uses the maximum value of the matched filter response as the threshold to obtain the binary image, and multiplies the binary image with the matched filter image. It is proved by the result that not only the segmentation results are accurate, but also the depth information of blood vessels could be displayed in different thicknesses, which is a new breakthrough compared with peers. Furthermore, we find that parameters of different kinds of blood vessels largely affects the performance of matched filter algorithm, while this problem has not been systematically discussed in other literature. Therefore, in this paper we establishes a model to help determine the optimum parameters of different kinds of blood vessels.

In this paper, we propose a vein imaging system based on a 2-D matched filter algorithm and implement it on an FPGA. Besides, the hardware structure is mainly composed of image sensor Complementary Metal-Oxide-Semiconductor (CMOS), infrared sources, Synchronous Dynamic Random Access Memory (SDRAM), Digital Light Projector (DLP), power supply and I2C protocols. The DLP module used in this project was OPD01M projection module produced by Ongnie Company. Its projection light source can be programmable controlled and can be set as RGB type. The imaging distortion is less than 1.0%, the uniformity of the imaging is greater than 85%, and the resolution of the display image is 752*480. The near infrared light source is a near infrared emitter, named SFH7252 produced by OSRAM. The wavelengths of near infrared ray (NIR) are 850nm and 940nm, and the power ratings are 140mW and 135mW.

The paper is organized as follows: Section 1 generally introduces the research background and basic framework. Section 2 clearly describes the methods and materials that we use, including the principle and design method of the key two-dimensional matched filters, and the hardware implementation on the FPGA. The experiment, evaluation, results and discussion are presented in Section 3. Finally, the conclusion and some valuable further work are presented in Section 4.

The main contributions of this article are as follows. First, we have completed a real-time vein imaging system based on matched filtering and established a mathematical model according to the blood vessels on the back of the human hand. This model can calculate the parameters related to the convolution kernel of the optimal matched filter according to the characteristics of the blood vessel image, which is also the basis for parameter selection in the whole subsequent paper. Secondly, our device has clinical value. Due to the thicker fat layer, smaller hand size and blood vessel diameter of newborn babies, it is more difficult to puncture than adults. This vein imaging device based on matched filtering algorithm has the potential advantage of being used to assist newborn with venipuncture (Figure 5 DEF).

**FIGURE 1.**
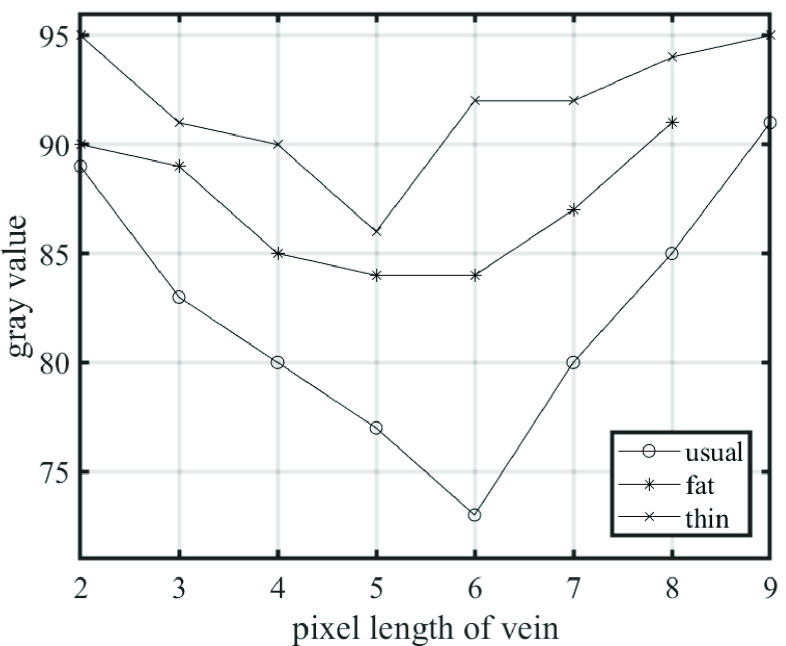
Gray level distribution of the cross section of the hand dorsal vein.

**FIGURE 2.**
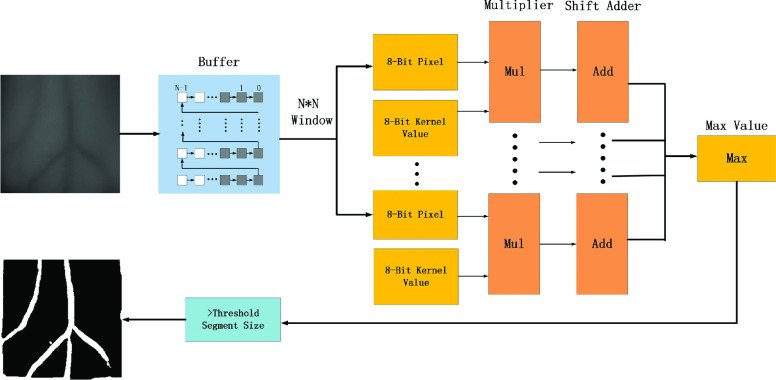
Process flow of the proposed algorithms.

**FIGURE 3.**
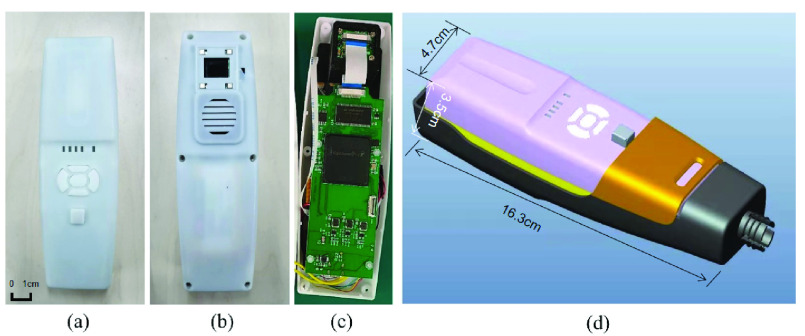
Prototype of the system: (a) front view; (b) back view; (c) front view without cover; (d) the overall system drawing.

**FIGURE 4.**
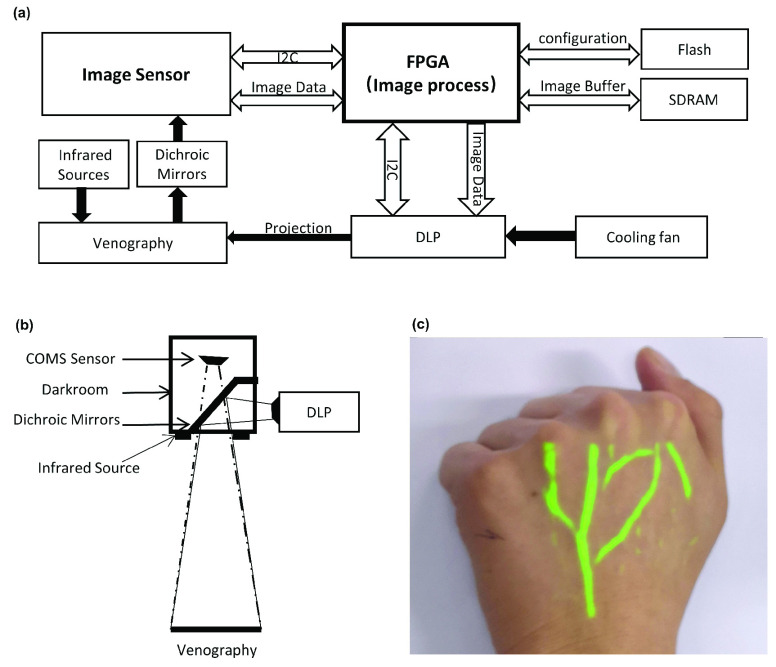
Framework of the proposed FPGA-based system: (a) whole hardware architecture, (b) multiplexing optical system of the DLP and CMOS, and (c) the actual application effect of this proposed vein imaging system.

**FIGURE 5.**
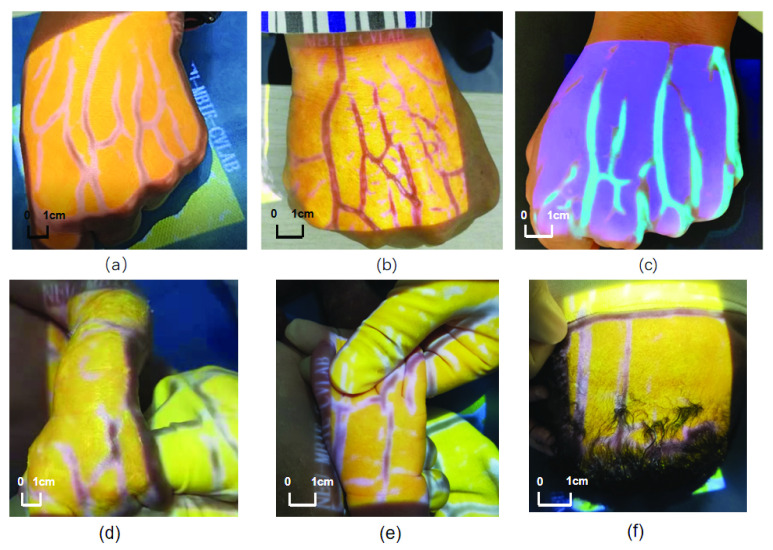
Different modes which can be transformed by buttons on the device (a) basic mode (b) enhancement mode (c) purple light mode, (d) (e) (f) Assist the newborn to perform puncture.

## II. Materials and Methods

### A. Matched Filter

The matched filter, which maximizes the ratio of instantaneous signal power to average noise power at the output, is a kind of optimal linear filter [16]. When applying a matched filter to detect an arbitrary signal s(t) corrupted by additive white Gaussian noise $\textrm {n}_{0} (t)$, the calculation and derivation of the instantaneous signal-to-noise ratio (SNR) will be described by Equation (1):

\begin{equation*} \rho _{0} =\frac {\left |{ {\textrm {y}(t_{0})} }\right |^{2}}{\overline {n_{0}^{2}(t)}}\tag{1}\end{equation*}

where $\textrm {y}(t_{0})$ can be obtained by inverse transformation of the spectrum density function (Equation (2)), where $H(\omega)$ is the transfer function of the filter:

\begin{equation*} \textrm {y}(t_{0})=\frac {1}{2\pi }\int \limits _{-\infty }^\infty {X(\omega)H(\omega)e^{j\omega t_{0}}d\omega }\tag{2}\end{equation*}

and the average power of the output noise can also be easily calculated as $\overline {\textrm {n}_{0}^{2}(t)} $. The representation can be simplified to Equation (3):

\begin{equation*} \rho _{0} \le \frac {2E}{n_{0}}\tag{3}\end{equation*}

Therefore, to maximize the SNR result, we need to use the equal sign.

### B. Two-Dimensional Matched Filter Algorithmic Design

Regarding the application in image processing, the essence of the matched filter algorithm is to design an optimal filter to match the shape of the object in the region of interest in the image [17]; and after the filtering process, the expected target can be separated from the original image with the most useful information.

By inspecting the gray-level profiles of the cross section of the hand vein, it is observed that the intensity profiles of these vessels have a characteristic inverted bell shape [18] that can be approximated by a Gaussian function. Meanwhile, since the shape of the optimal filters must match the shape of the vessel to the greatest extent, the mathematical expression of this optimal filter is defined as Equation (4):

\begin{equation*} K(x,y)=-e^{-\frac {x^{2}}{2\sigma ^{2}}},\quad \left |{ x }\right |\le 3\sigma,\left |{ y }\right |\le \frac {L}{2}\tag{4}\end{equation*}

where $K(x,y)$ is called the kernel function; coefficient $\sigma $ defines the degree of deviation of the Gaussian function from the center of the X-axis, that is, the width of the vessel in this application; and $L$ is the length of the blood vessels truncated along the Y-axis. Among them, the selection of the coefficient $\sigma $ is based on the gray level difference of the blood vessel cross section. Therefore, different groups of people should choose different $\sigma $ (Figures 5 and 11).

**FIGURE 6.**
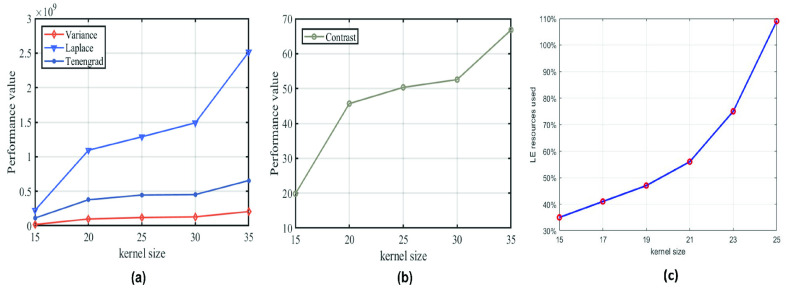
Quantitative analysis result of parameter selection: (a) indicators of sharpness and smoothness: Tenengrad, variance and Laplace smoothness; and (b) indicators of contrast; and (c) The relationship between Logic Elements (LE) in FPGA and convolution kernel size.

**FIGURE 7.**
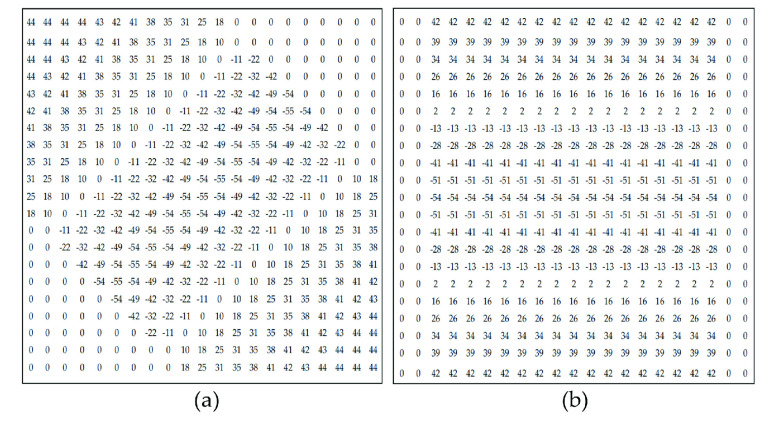
(a). A $21\times 21$ matched filter with $\sigma =3.3$ and $L=16$ when $\theta = 45^{\circ }$ (b). A $21\times 21$ matched filter with $\sigma =3.3$ and $L = 16$ when $\theta = 0^{\circ }$.

**FIGURE 8.**
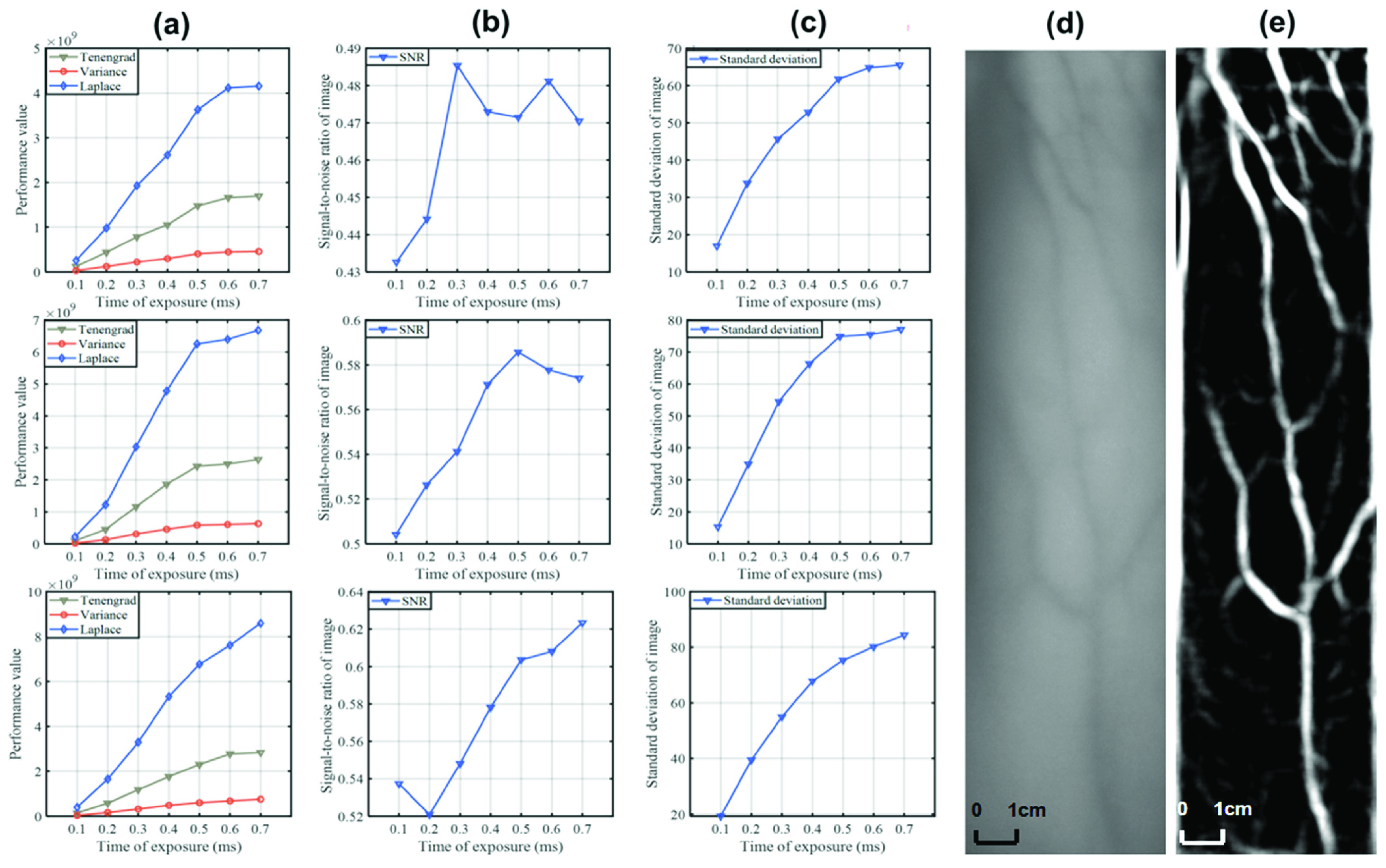
Quantitative analysis of the influence of the exposure duration (a) Sharpness and smoothness values of the matched filter output under different exposure durations. (b) Signal-to-noise ratios of the matched filter output under different exposure durations. (c) Standard deviations of the matched filter output under different exposure durations. (d) Original image. (e) Matched filtered output under an exposure time of 0.6ms.

**FIGURE 9.**
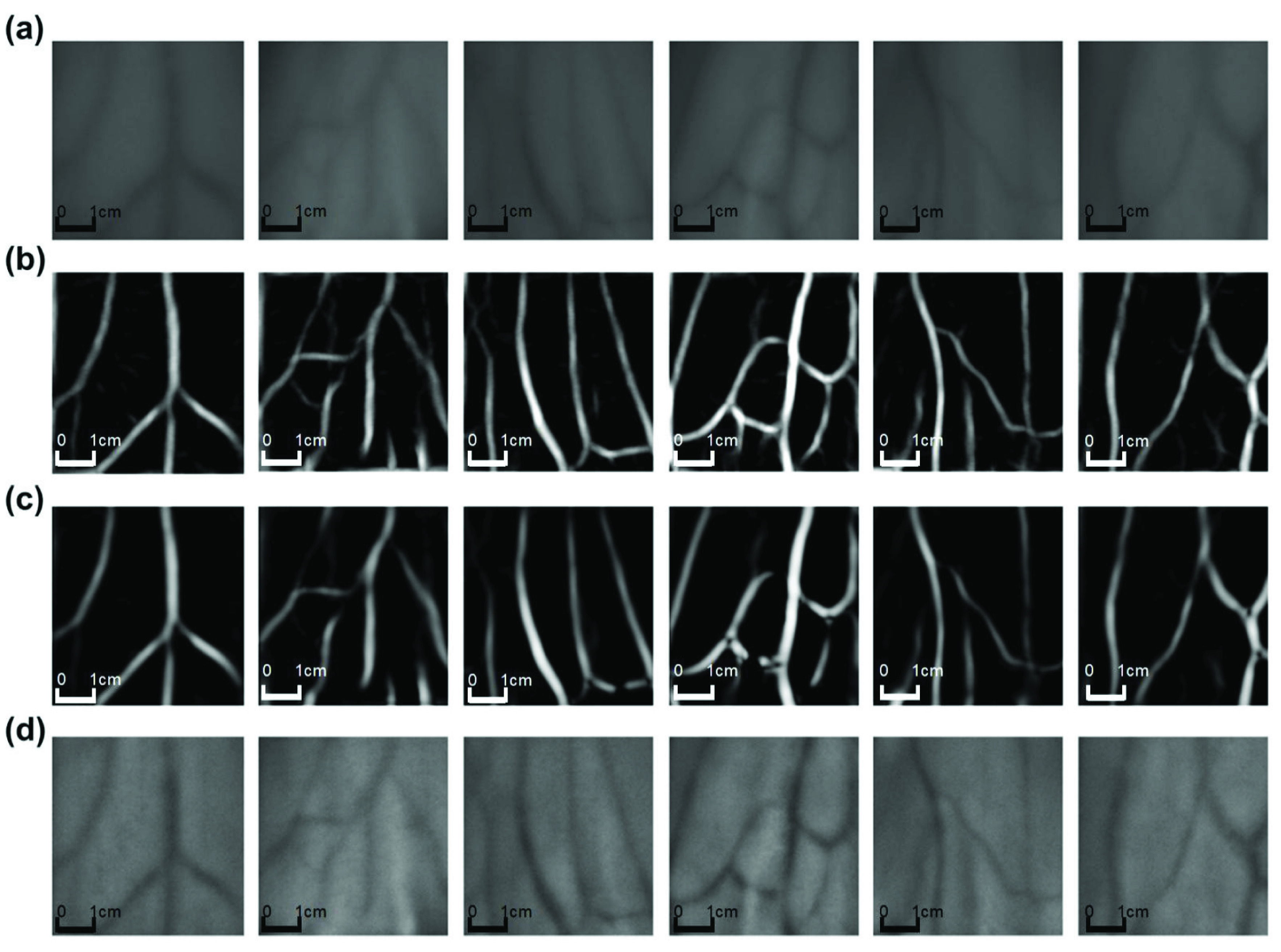
Comparison of the original images and the results of 3 different algorithms: (a) original image of the vein area, (b) results of the proposed matched filter, (c) results of Frangi image segmentation, and (d) results of CLAHE image segmentation.

**FIGURE 10.**
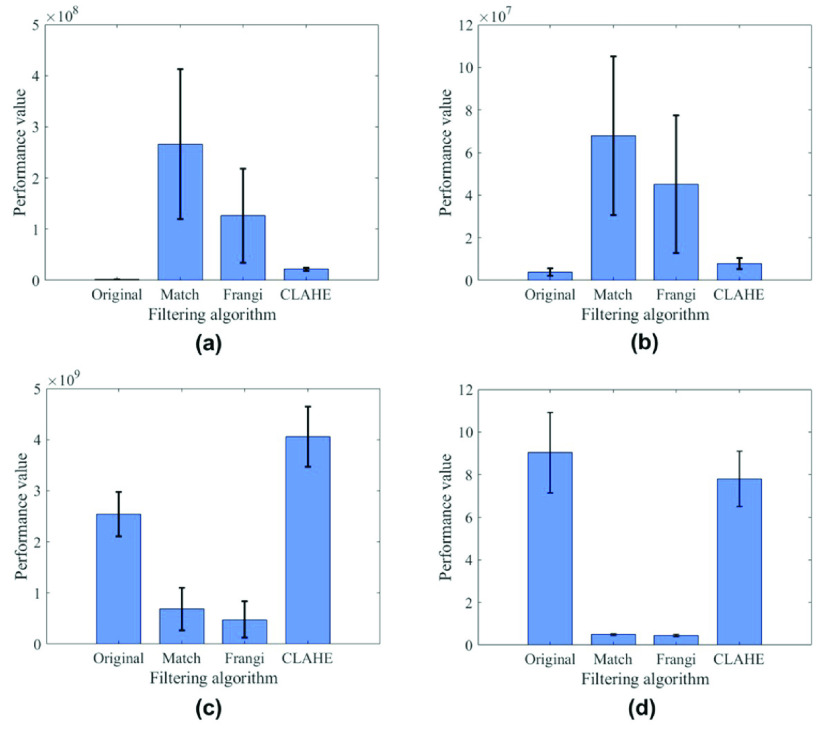
Performance comparison of different algorithms based on the datasets mentioned in the paper. Mean values of the (a) Tenengrad, (b) variance, (c) Laplace smoothness, and (d) SNR from the original image and filtered image. Vertical bars represent the standard errors of the mean.

**FIGURE 11.**
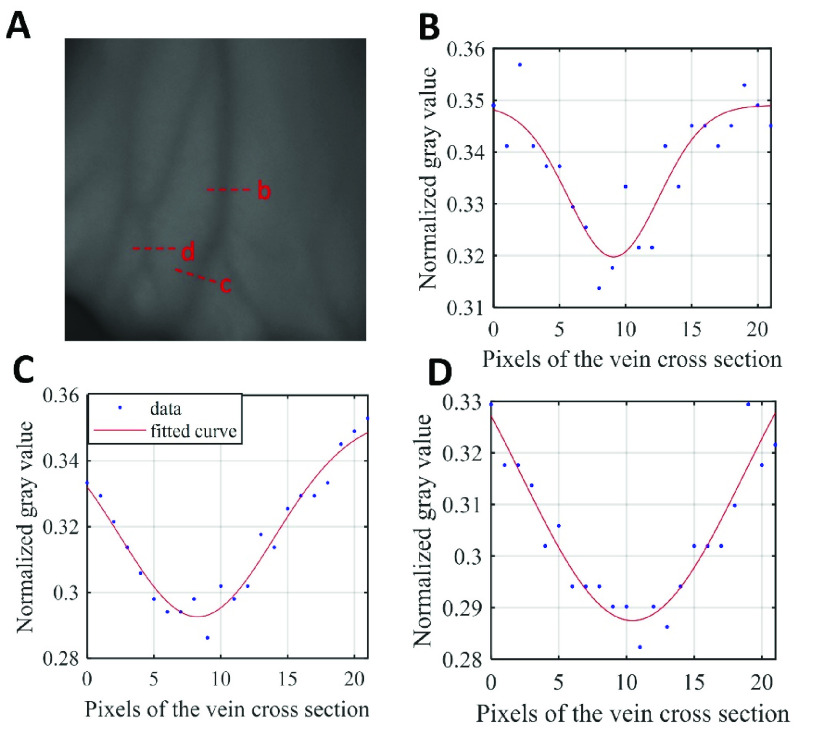
Gaussian fitting curve of veins with different thickness: (A) Vein image; (B) Fitting curve of vein cross section b in figure A, $\sigma = 3.434$; (C) Fitting curve of vein cross section c in figure A, $\sigma = 5.742$; (D) Fitting curve of vein cross section d in figure A, $\sigma = 8.157$.

In this algorithm, we design vascular like templates representing different directions as filters to match the hand vein images. It is simply appreciated that a vessel may be oriented at any angle $\theta (0\le \theta \le \pi)$; thus, our Gaussian curve can rotate to match the blood vessels in different directions. The number of filters n is determined by the following formula: $\textrm {n}(\theta)=180/\theta $, where $\theta $ represents the angular resolution of the matched filters. The angular resolution and size of the matched filters are related to the filtering performance and the cost of hardware resources. Therefore, considering both factors simultaneously is important. By verifying the pre-experiments in MATLAB and the simulation trials on an FPGA, we find that the best performance along with appropriate low cost appeared when using 4 matched filters. That is, the angular resolution is 45°.

First the filter template rotates at different angles should be calculated. Let $\textrm {p}=\left [{ {x,y} }\right]$ be a discrete point in the kernel and $\theta _{i} $ be the orientation of the $i$th kernel matches to a vessel at angle $\theta _{i} $. The Gaussian kernels are assumed to be centered about the origin [0, 0] when calculating the weighting coefficients. The rotation matrix is given by:

\begin{align*} R_{i} =\left [{ {{\begin{array}{cc} {\cos \theta _{i}} & {-\sin \theta _{i}} \\ {\sin \theta _{i}} &{\cos \theta _{i}} \\ \end{array}}} }\right]\tag{5}\end{align*}

The corresponding point in the rotated coordinate system is given by $p_{i} =\left [{ {u,v} }\right]=pR_{i} $. Since the Gaussian function is a curve with infinite length along both the positive and negative directions of the X-axis, in the whole dorsal image only the gray distribution of the cross-section of the hand vein is consistent with it; therefore, the Gaussian function that is used to match hand veins needs to be truncated. Based on the experience of Chaudhuri et al [17], we defines a neighborhood $N$, which is represented as:

\begin{equation*} N=\left \{{{(u,v)\left |{ {\left |{ u }\right |\le 3\sigma,\left |{ v }\right |\le \frac {L}{2}} }\right.} }\right \}\tag{6}\end{equation*}

Accordingly, the rotated weighting coefficients in the $i$th kernel can be represented by $u$ and $v$, which are the new coordinates after rotation.

If the number of points in area $N$ is denoted by $A$, the mean value of the kernel is determined as Equation (7):

\begin{equation*} m_{i} =\frac {1}{A}\sum \limits _{p_{i} \in N} {K_{i} (x,y)}\tag{7}\end{equation*}

### C. Hardware Architecture of the Proposed Matched Filter

Two-dimensional matched filtering is a template-based image processing technology, whose key operation is the convolution between pixels in the image window and the convolution kernel template. The convolution kernel template is calculated according to the algorithm described in section 2.2. The filter coefficients of these filters are calculated in MATLAB in advance, and these precalculated 8-bit convolution kernel weights are used as macro definitions in the Verilog language under the Quartus II environment.

Based on the parallel characteristics of the multiplication and addition process in the convolution operation and the pipeline design of the field programmable logic gate array (FPGA), the implementation of the two-dimensional matched filtering algorithm on the FPGA can be divided into two modules: the image data cache, namely, the I/O module; and the convolution operation module. The hierarchical design of the FPGA module is similar to a system proposed by Koukounis et al [19]; however, our designed hardware execution process is simpler, saves more hardware resources, and is more efficient.

#### 1) Image Data Cache and $I/O$ Module

The I/O module is mainly responsible for processing the pixel data to make them input the image processing flow in parallel and administer the output of the pixel value after filtering. This part is separated from the subsequent calculation operation module, therefore the convolution operation between multiple 2D matching filter templates and image windows can be conducted in parallel at the same time, so as to improve the execution efficiency. First in first out (FIFO) is used as the row buffer of the image data. We can see from the first flow of figure 2 that N-1 row buffers are used for the $N\times N$ image window to cache the serially-read image data and convert the N-line pixel value to parallel output.

However, due to the complexity of the read and write operations of FIFO, this module can be optimized by using the shift register macro module altshift-taps based on RAM to improve the image data line buffer function.

#### 2) Parallel Pipeline Convolution Module

According to the two-dimensional matched filtering the algorithm designed above, we use a filter bank consisting of 4 two-dimensional matched filters, with an angular resolution of 45° as convolution templates. In the convolution module, the parallel image data output by the I/O module will conduct convolution operation with different filters at 0°, 45°, 90° and 135° respectively. We calculate these 4 matching filters in MATLAB, and the original values obtained are decimals less than 1. In order to make them calculate in FPGA, we take the first two decimal places and multiply them by 100. Then these template coefficients obtained are used in convolution.

Considering the feature of multi angle rotation, flexible and portable of matched filter templates, there is no uniform numerical symmetry relationship when calculating convolution, so it is needed to calculate the one-to-one correspondence multiplication of template coefficients and window pixels. According to original value of the coefficients, eight bits signed binary numbers is enough to represent a coefficient in the template. Therefore, all $N^{2}$ coefficients in a template can be represented by an $N^{2\ast}8$-bit number. Image pixels can also be represented by such a number and multiply the corresponding positions. The multiplication in convolution is realized by multiplying the corresponding phase of these two $N^{2\ast} 8$-bit numbers.

In convolution operation, if the multiplier is directly used to multiply the image window pixels and template coefficients, it will consume a large amount of hardware resources. Therefore, we use shift addition to improve the resource utilization efficiency. Shift addition is a simple multiplication method that is similar to manual calculation. Starting from the lowest bit of the multiplier, when the bit is 1, the multiplier is shifted i bits to the left and then added to the previous sum; and when the bit is 0, the multiplier is shifted i bits to the left and then 0 is added to the previous sum. The greatest advantage of the shift adder is that it saves resource, which is appropriate for FPGA design. There are also some other methods to realize the multiplication and addition operations in the convolution operation [18], [19], although shift addition performs optimally when implementing large-scale multiplication, such as this application in matched filters.

Result value of the above multiplication is a $N^{2}\times 16$-bit number. And we need to add all the product values to calculate the convolution. Divide this $N^{2}\times 16$-bit number by two to get the front and back half, extract the first 16 bits from the two numbers each time and add them, until the length of the two numbers is 0. So we get the final convolution result. From the four filters, namely, the convolution result values in the four directions, the largest value is selected as the result value of the matched filtering in the present window. Windows that have the same size as the proposed filters traverse the entire image to complete FPGA-based parallel convolution.

#### 3) Composition of the Total System

The proposed vein imaging system adopts an FPGA as the core processing architecture. The actual product is shown in Figure 3, and the size of this integrated system is 16.3*4.7*3.5. Figure 4 (a) briefly displays the hardware design of our system. The infrared image of the vein illuminated by the infrared light source is captured by the image sensor through the dichroic mirror. The FPGA communicates with the image sensor through the I2C-bus specification, and then fetches the image data captured by the image sensor through the parallel port. After the core matched filter processing within the FPGA, the image is cached in the SDRAM. The FPGA controls the transmission of the video stream, and the processed image is transmitted to the DLP and then projected to the original position of the collected image for display. Figure4(b) describes the internal structure of the image sensor CMOS. It is sealed in a dark room and captures 850 nm and 940 nm infrared optical images reflected by the skin through a dichroic mirror. The design of the darkroom greatly ensures the accuracy of infrared optical images and filters out interference caused by visible light to a large extent. The optical path adopts a noncoaxial design. That is, the projector and the image sensor are not on the same axis. The infrared light source is a package that combines 850nm and 940nm emission tubes. Four infrared light sources are centered on the camera to ensure uniform illumination, which are irradiated on the skin and refracted to generate infrared images. Figure 4 (c) shows the actual application effect of this proposed vein imaging system.

In order to ensure that the image projected by DLP is aligned with the actual position of blood vessels in the human body, our system designs an image movement module. The principle of this module is to control the line sync signal and the vertical sync signal with the key to achieve the translation of the image in all directions in the projection.

In order to improve the clinical applicability of the system, we design four projection modes for the device, namely basic mode, enhancement mode, green light children mode for the thinner blood vessels and purple light mode. These four different modes can be switched through buttons on the device, and their actual application effect is shown in figure 5 below.

## III. Results and Discussion

In order to determine the requirements of the targeted FPGA, we implement the experimental architecture on a Cyclone IV EP4CE115F29C8 FPGA. Table 1 below gives the analysis and synthesis results for the entity system implemented on the FPGA. The table lists the overheads of FPGA slice source, such as total elements, total registers, and the total memory bits and pins we distribute in order to give a complete illustration of the required source.

**TABLE 1:** Analysis and Synthesis Results for the Entity System Implemented on the FPGA

Platform	Total logic Elements(76028)	Total Registers (66235)	Total memory bits(185093)	Total pins (117)	Freq. (MHz)
Cyclone IV EP4CE115F29C8	67 %	–	5 %	22 %	50

A wide variety of experiments have been conducted to verify the excellent performance of this proposed FPGA-based 2-D matched filtering design. We initially compare its performance with other hand vein segmentation methods according to several different indicators, and conclude that the proposed design performed the best. Besides, for this special programmable hardware architecture, we find that the variability of the light intensity of the LED and the parameters of the convolution template contribute to different effects, so a separate analysis is performed.

### A. Dataset and Measures

We used the proposed system to capture 242 images including 235 dorsal images and 7 wrist images from 120 participants and constructed our customized vein image dataset. The size of these images is $752\times 480$ pixels. According to physical differences among these images, we divided the total images into 4 groups: thick, thin, fat and winding. All the following experiments were implemented on this dataset.

The performance of the proposed system is measured and compared according to different indicators, which are the Tenengrad evaluation function, variance, Laplacian operator [20]. Among these metrics, the Tenengrad evaluation function and variance are indexes of image clarity. The Laplacian operator mainly evaluates the smoothness of the image. Tenengrad is a commonly used image sharpness evaluation function based on gradients. It utilizes the Sobel operator to extract the horizontal and vertical gradient values, and the sum of squares is used as the evaluation function. Usually, the images with better focus have sharper edges, so they have larger gradient function values. That is, the sharper image has the larger Tenengrad value. The Laplacian operator is used to measure the smoothness. The calculation result is obtained by squaring the sum of the second derivative in the horizontal and vertical directions. That is, it was calculated by:

\begin{align*} \psi =\sum \limits _{i=0}^{m-1} {\sum \limits _{j=0}^{n-1} {\left [{ {\begin{array}{l} f(i+1,j)+f(i-1,j) \\ + f(i,j+1)+f(i,j-1)-f(i,j) \\ \end{array}} }\right]}}^{2}\tag{8}\end{align*}

It is clear that a smaller value of the Laplacian operator $\psi $ means better smoothness. Besides, the SNR and variance are also selected as image quality evaluation metrics.

### B. Parameter Selection

#### 1) Parameters of the Filter

The key algorithm we use in this vein imaging system is 2-D matched filtering. Its essence is matching the intensity distribution of the blood vessel cross section with a rotated Gaussian function; therefore, it has 3 alternative filter parameters including the size of filter, $\sigma $ and $L$ whose values need to be adjusted according to the concrete conditions of the participants’ veins.

Different sized matched filters are used to process the above dataset, and the sharpness, smoothness and contrast of the filtered images are calculated. The results are shown in figure 6 below.

The figure 6 shows that when the size of the convolution kernel increases from 15 to 20, the sharpness and contrast obviously improve. When size of the convolution kernel is 20–30, the sharpness and contrast tend to be stable, and then increase again when the size is 30–35. If the size is $35\times 35$, the contrast is the largest, so the extracted blood vessels are the most obvious. However, some noise points will be extracted as blood vessels. In addition, the consumption of FPGA internal resources increases with the increase of the convolution kernel window. When the window size is opened above 21, the consumption of logic resources will increase significantly, so under the dual factors of hardware internal resources and image performance, choosing the size of the convolution kernel to 21 is the optimal solution.

Therefore, we apply a series of $21\times 21$ rotated Gaussian filters among all the follow-up experiments. Two examples of our rotated filters that represent filter at 45° and 0°, respectively, are displayed in figure 7.

Differences in the physical properties of blood vessels cause differences in the optimal fitting parameters. For instance, a fat vein may have the best performing $\sigma $, which differs from that of a thin vein. The curve fitting application in MATLAB is utilized to obtain the optimal fitting parameters $\sigma $ for the Gaussian cross section of the intensity images. Thin veins have small cross-sectional grayscale differences, so the $\sigma $ is high, on the contrary, the $\sigma $ of the thick veins is low (Figure 11). According to the corresponding relationship between the thickness of the vein and $\sigma $, we apply different $\sigma $ parameters to filter for adults (Figure 5 ABC) and newborns (Figure 5 DEF).

#### 2) Effect of Exposure Duration

The image quality obtained by the CMOS sensor has a great impact on the subsequent vein filtering and segmentation. Specifically, exposure time is an important parameter during shooting, which affects the image details, namely the acquisition of small vessels. Therefore, we conduct a set of experiments to determine the best exposure time for the camera to take vein images. When the light intensities of 850 nm and 940 nm infrared light sources are both 50 mW, the image quality after matching filtering is analyzed by changing the exposure time.

Generally speaking, the selection method of exposure duration is to maximize the dynamic range of the image. In this experiment, we find that the dynamic range of the image under different exposure intensity is 0–255. Therefore, we use Tenengrad, variance, Laplace objective evaluation index to determine the exposure duration (Figure 8). From the experimental results, as the exposure time increases, the quality of the captured image also increases. When the exposure time reaches 0.7 seconds or more, the image will be overexposed. In the obtained image, the reflected light from the light source to the skin epidermis is far greater than the reflected light inside the skin, and the collected image has relatively large noise, which affects the image collected in the original light path. At the same time, when the exposure time is increased from 0.1ms to 0.6ms, the image quality is improved greatly, but when the exposure time is greater than 0.6ms, the increase in image quality is not significant, so in this device, the exposure time of 0.6ms is reasonable.

### C. Performance Result

Contrast limited adaptive histogram equalization (CLAHE) is a commonly used method for enhancing the regional contrast in low contrast images [21]. Its principle is slightly difficult and complex to fulfill, although it works great. Another classical comparison method we select in the experiment was proposed by Frangi et al [22]. They speculated on the presentation of vessels in the likelihood by utilizing the eigenvalues of the Hessian. We cut the image size to $211\times 211$ and run these 3 methods on the same customized dataset in MATLAB, and obtain the resulting images displayed in figure 9. It can be clearly seen that the proposed algorithm greatly segments the shape of vein, and the depth information is obvious.

It can be seen from the resulting values that the proposed design generally has great performance. The result is shown as figure 10, both the matched filtering algorithm and the Frangi algorithm have very good performance. In the sharpness test experiment, it can be known that the clarity of the image calculated by the matched filtering algorithm under the Tenengrad index exceeds 2 times that of the image processed by the Frangi algorithm. Also under the Variance indicator, the performance of matched filtering is 3/2 times that of the Frangi algorithm. In terms of image signal-to-noise ratio, the performance of the two algorithms is the same. In the experiment of the smoothness test, the image processed by the Frangi algorithm has better smoothness than the image processed by the matched filter, and the smoothness index of Frangi algorithm is 29.9% lower than that of matched filter algorithm. At the same time, the time complexity of the Frangi algorithm is greater than that of matched filtering, and it is difficult to process images in real time. Therefore, the matched filtering algorithm has a great advantage in blood vessel segmentation.

The proposed FPGA-based matched filter algorithm has achieved promising processing speed. We calculate the processing speed of the algorithm under the FPGA pipeline processing mechanism. The multiplication needs to consume 4 clocks, the subsequent addition needs to consume 9 clocks, and the final extraction maximum needs to consume 2 clocks. Therefore, when a pixel enters the image processing module until the final processing is completed, 15 clock resources are consumed. For a frame image of 752*480, 360974 clocks are needed to completely process the image. The CMOS camera pixel clock used in the project is 24MHz, so it takes 0.015 seconds to process an image. It is easy to calculate that the image processing speed can reach 67 frames per second.

We compare the processing time and speed of several related matched filtering algorithms. Comparison result are shown in the table 2 below. It can be seen that the execution time namely processing speed of FPGA-based two-dimensional matched filter algorithm for hand vein imaging has a significant advantage.

**TABLE 2:** Comparison Between Related Matched Filtering Algorithms With the Proposed System

	Frequency (MHz)	Execution time(s)	Platform	Algorithm	Image resolution	Convolution window size
Chaudhuri et al[17]	N/A	2.0000	Special Image Processing board	MFR	$512\times 480$	$32\times 32$
Hoover et al [11]	N/A	3.0000	Sun SPARC Station 20	MFR+Threshold Probing	$700\times 605$	$16\times 16$
Dimitris et al [13]	100	0.03139	FPGA	MFR+Thresholding	$768\times 584$	$15\times 16$
Proposed Architecture	24	0.01504	FPGA	MFR+Thresholding	$752\times 480$	$21\times 21$

## IV. Conclusion

In this paper, we propose a vein imaging system based on an FPGA chip, which can realize the complex algorithm of matched filtering, effectively extract the blood vessel part and retain the depth information of the blood vessel. The FPGA can also take advantage of its pipeline parallel computing to ensure the real-time image display performance. Furthermore, this paper also assesses the exposure time when taking blood vessel images, determines the optimal convolution kernels for the blood vessel algorithm and performs an image quality assessment as a quantitative analysis. It is concluded that under the premise of guaranteeing sufficient FPGA resources, $21\times 21$ convolution kernels, $L=16$ and $\sigma =3.3$ can achieve optimal blood vessel extraction, and the matched filtering algorithm is better than the previously proposed CLAHE image segmentation method and the Frangi multi-scale image segmentation method. Besides, the biggest advantage of this proposed design is the adjustable-parameters. Therefore, in the field of embedded image processing, this system is considered as a vein imaging system with excellent performance, which can provide a reference for hardware architecture and software algorithm in the field of vein imaging.
